# Feigning Adult ADHD on a Comprehensive Neuropsychological Test Battery: An Analogue Study

**DOI:** 10.3390/ijerph20054070

**Published:** 2023-02-24

**Authors:** Miriam Becke, Lara Tucha, Marah Butzbach, Steffen Aschenbrenner, Matthias Weisbrod, Oliver Tucha, Anselm B. M. Fuermaier

**Affiliations:** 1 Department of Clinical and Developmental Neuropsychology, University of Groningen, 9712 TS Groningen, The Netherlands; 2 Department of Psychiatry and Psychotherapy, University Medical Center Rostock, Gehlsheimer Str. 20, 18147 Rostock, Germany; 3 Department of Clinical Psychology and Neuropsychology, SRH Clinic Karlsbad-Langensteinbach, 76307 Karlsbad, Germany; 4 Department of Psychiatry and Psychotherapy, SRH Clinic Karlsbad-Langensteinbach, 76307 Karlsbad, Germany; 5 Department of General Psychiatry, Center of Psychosocial Medicine, University of Heidelberg, 69115 Heidelberg, Germany; 6 Department of Psychology, National University of Ireland, W23 F2K8 Maynooth, Ireland

**Keywords:** attention-deficit/hyperactivity disorder, neuropsychological assessment, performance validity, symptom validity, noncredible performance, feigning

## Abstract

The evaluation of performance validity is an essential part of any neuropsychological evaluation. Validity indicators embedded in routine neuropsychological tests offer a time-efficient option for sampling performance validity throughout the assessment while reducing vulnerability to coaching. By administering a comprehensive neuropsychological test battery to 57 adults with ADHD, 60 neurotypical controls, and 151 instructed simulators, we examined each test’s utility in detecting noncredible performance. Cut-off scores were derived for all available outcome variables. Although all ensured at least 90% specificity in the ADHD Group, sensitivity differed significantly between tests, ranging from 0% to 64.9%. Tests of selective attention, vigilance, and inhibition were most useful in detecting the instructed simulation of adult ADHD, whereas figural fluency and task switching lacked sensitivity. Five or more test variables demonstrating results in the second to fourth percentile were rare among cases of genuine adult ADHD but identified approximately 58% of instructed simulators.

## 1. Introduction

A considerable minority of examinees underperforms in neuropsychological assessments due to a wide array of possible reasons, including maladaptive responses to genuine health conditions and the exaggeration or feigning of symptoms [1]. The evaluation of symptom and performance validity is therefore an important cornerstone in strengthening the quality of clinical data as well as the conclusions derived from clinical assessments. As such, the continuous sampling of symptom and performance validity throughout neuropsychological examinations is now widely supported [2,3,4]. Symptom validity may be assessed via rating scales completed by either observers or examinees themselves (i.e., symptom validity tests (SVTs)). On the other hand, stand-alone performance validity tests (PVTs) have been developed for the explicit purpose of detecting noncredible performance in neuropsychological assessments [5,6,7] and are typically considered the most sensitive instruments serving this purpose [8]. However, continuous sampling of performance validity across cognitive domains necessitates the use of multiple freestanding PVTs, a practice complicated by significant increases in test-taking time and the fact that many standalone PVTs are memory based [9]. Since practitioners frequently face pressure to shorten assessments and maximize cost-efficiency, the expansive use of stand-alone PVTs may not be feasible.

Due to their favorable time efficiency and low face validity [10,11,12,13], validity indicators embedded in routine neuropsychological performance tests may be more suitable for the ongoing monitoring of performance validity than their stand-alone counterparts. Embedded validity indicators (EVIs) offer the advantage of measuring clinically relevant constructs: If an examinee’s performance is deemed valid, the test on which the EVI is based offers clinically relevant information about their cognitive status in the assessed domain. At the same time, this effect entails a disadvantage inherent to EVIs. They are more sensitive to cognitive impairment and consequently require ample validation to prevent genuine impairment from being mistaken for invalid performance (see invalid-before-impaired paradox, [14]). This limitation may be overcome by combining multiple performance validity indicators [15,16].

The high base rates of symptoms associated with the condition, various incentives, and the potentially far-reaching consequences of false positive diagnoses make the evaluation of symptoms and performance particularly pertinent to the assessment of ADHD. Motivated by the prescription of stimulant medication, access to accommodations, or social incentives, examinees have been shown to feign or exaggerate symptoms of ADHD convincingly with relative ease [7,17,18,19,20,21,22,23,24,25,26]. Expectations of improved cognitive functioning and thus increased study or work performance, the intent to use substances recreationally, or to distribute medications on the black-market motivate some to seek an evaluation of ADHD. Others may hope for accommodations such as additional time for assignments or exams, adapted forms of testing, or assisting technology (e.g., noise-cancelling headphones). Recently, attention has further been drawn to incentives pertaining to social situations: an unwarranted diagnosis of ADHD may provide explanations for academic underachievement or social mishaps, such as being late, forgetful, inattentive, or impulsive [27].

The consequences of false-positive diagnoses are potentially severe, both at the individual and the societal level. Superfluous treatment with stimulant medication puts people at risk of adverse events. Scarce resources, including the previously mentioned assisting technology, may not be adequately allocated as a consequence of unwarranted diagnoses, and false-positive diagnoses violate the principle of equal opportunity in academic settings. Furthermore, they may undermine the public perception of ADHD. Including feigned cases of ADHD in research studies could ‘dilute’ samples and therefore contribute to heterogeneous findings.

Although no single distinct cognitive profile can be associated with ADHD [28,29,30] and, therefore, neuropsychological assessments are neither sufficient nor required to reach a diagnosis, neuropsychological performance tests help quantify subjective impairments, evaluate treatment options, and chart the course of the disorder. Aspects of attention and executive functioning have been variably implicated in ADHD, with domains such as working memory, response inhibition, and vigilance frequently showing impairments [30,31,32,33]. Various tests assessing these commonly affected domains have been examined with regard to their utility as potential EVIs. Among these, tests of attention are promising options, since impairments of attention are common and therefore frequently assessed in populations with neurological or psychiatric conditions [33,34,35,36].

Continuous performance tests (CPTs), for example, are among the most commonly used neuropsychological tests [37] and the most extensively researched EVIs. Their outcome variables usually include (hit) reaction times and their variability, alongside errors of omission and commission; the latter two of which are conceived to indicate inattention and impulsivity, respectively. This inclusion of both the speed of response parameters and measures of accuracy has been considered a strength for tests of attention, including CPTs [38]. Using different CPT versions, including Conner’s Continuous Performance Test (CPT-II) [39] and the Test of Variables of Attention (TOVA) [40], all four of these outcome measures have shown some utility in detecting invalid performance in mixed clinical samples [15,23,41,42,43,44,45,46,47,48,49,50]. Ord et al. [51] found that the dispersion of hit reaction times and the change in interstimulus intervals associated with hits are the strongest predictors of PVT failure in a sample of veterans, followed by errors of omission and commission. Due to low sensitivity, however, the authors caution against the isolated use of these EVIs. Similarly, Scimeca et al. [52] concluded that individual outcome variables of the CPT-3 [53] lacked classification accuracy, particularly a low sensitivity if adequate specificity was to be ensured, and advised against its isolated use in samples under evaluation for possible ADHD. Fiene and colleagues [54] further noted that the utility of reaction time variability found in CPTs could also be observed in a simple reaction time task, a finding later independently reported in other studies [55,56]. Variations of the Stroop Test [57] have also shown promising results across studies [47,58,59,60,61,62,63], with the word reading trial oftentimes emerging as the most sensitive outcome measure within this test.

Findings on other tests have been more heterogenous, particularly with regard to sensitivity. The Processing Speed Index (PSI), measured as part of the Wechsler Adult Intelligence Scale-IV (WAIS-IV) [64], has shown variable sensitivity and may put populations with cognitive impairments at risk of false-positive classifications [23,41,65,66]. Its overall acceptable classification accuracy has lent support to the use of the Trail Making Test (TMT) [67] as an adjunct marker of performance validity [23,43,47,61,68,69,70,71], yet its variations have yielded varying estimates of sensitivity across studies. Complex variations of digit spans (e.g., RDS-WM using the WAIS-IV) [64,72] measuring working memory, detected invalid performance in the assessment of ADHD with adequate accuracy in a study conducted by Bing-Canar and colleagues [73]. Verbal fluency tasks have been found to be able to discern valid from invalid performance [61,74], though some studies report low sensitivity and concerns about classification accuracy among people with cognitive impairment [61,75].

The wide array of available instruments has complicated efforts to estimate the base rate of noncredible performance in the assessment of adult ADHD and to characterize examinees who fail PVTs. Early studies, which commonly used a single PVT to evaluate performance validity, reported base rates between 15% and 48% [76,77,78]. Later studies often applied a stricter criterion of two or more PVT failures across a battery of tests and found base rates ranging from 11% to 19% [7,79,80,81,82]. These later estimates resemble those of neuropsychologists in clinical practice, who approximate the base rate of noncredible performance in the assessment of adult ADHD to be around 20% [83]. Indeed, several studies have noted base rates converging around 20%, although some describe rates as high as 50% [77,78,84].

We administered an extensive neuropsychological test battery to adults with ADHD, neurotypical controls, and instructed simulators to examine their use as PVTs. Our primary aim was to develop cut-off scores for various outcome variables yielded by the tests in our battery and determine their accuracy in detecting the instructed simulation of adult ADHD. Since the battery included numerous tests in addition to a CPT, we were able to provide additional insights into the classification accuracy of multiple embedded performance validity indicators considered jointly (i.e., cut-off for the number of failures across a complete test battery). Moreover, it allowed us to examine possible changes in simulation effort throughout a comprehensive assessment. That is, whether instructed simulators show invalid performance throughout the assessment or on specific tests only and whether simulation efforts remain stable or fluctuate throughout the completion of said test battery.

## 2. Materials and Methods

### 2.1. Participants

Two approaches were used to recruit participants: first, 247 students enrolled in undergraduate psychology courses at the University of Groningen participated in exchange for course credit. Second, archival data collected as part of the clinical assessment of 73 adults diagnosed with ADHD were made available to the Department of Clinical and Developmental Neuropsychology at the University of Groningen.

#### 2.1.1. ADHD Group

Seventy-nine adults were initially considered for inclusion in the ADHD Group. Among them were archival data of 73 individuals with suspected ADHD, who had been referred to the Department of Psychiatry and Psychotherapy at the SHR Clinic Karlsbad-Langensteinbach for their clinical evaluation, as well as six additional participants from the student sample who presented with secured diagnoses of ADHD.

As a specialized outpatient clinic, the SRH clinic provides comprehensive diagnostic workups to adults whose general practitioners, psychiatrists, or neurologists suspect the presence of ADHD but do not believe they are sufficiently experienced or qualified to diagnose the disorder in adulthood. Despite the fact that all participants in this group experienced ADHD symptoms and impairments throughout childhood and adolescence, reliable information on a formal diagnosis of ADHD having been made in childhood could not be retrieved for all cases. Consequently, the diagnostic procedure followed the criteria for first-time adult ADHD diagnoses e.g., [85].

With participants having been informed that their participation in this study would not affect their clinical evaluation or treatment, this diagnostic procedure was undertaken by two experienced professionals who conducted extensive clinical interviews based on the *Diagnostic and Statistical Manual of Mental Disorders* [86]. Corroborating evidence of ADHD-related impairments was also gathered, whenever accessible, by asking parents, partners, and/or employers about difficulties observed at school, home, or work. Academic underachievement, negative teacher evaluations, unstable employment histories, financial problems, frequent relationship break-ups, repeated legal incidents, and poor driving records all provided objective evidence of impairment. Retrospective accounts of ADHD-related symptoms and impairments experienced during childhood and adolescence are required for a first-time diagnosis of the disorder in adulthood, and no formal diagnosis was made in the absence of such evidence. The examinees also completed standardized self-report measures of past and present ADHD symptoms (reported in Table 1), as well as a performance validity test.

Diagnoses of ADHD could ultimately not be confirmed for 16 participants, who were consequently excluded from the present study. Five participants were further excluded due to incomplete (n = 1) or failed (n = 4) validity tests (i.e., missing or suspect results on the TOMM; see Materials). Missing data on the neuropsychological test battery led to the exclusion of one additional participant. A summary of the demographic data for the remaining 57 adults with ADHD can be found in Table 1. Demographic data included a quantitative description of ADHD symptoms in the present and the past, which confirmed high levels of experienced ADHD symptomatology. The combined symptom presentation was most common (n = 30, 52.6%) in this final sample, followed by the inattentive symptom presentation (n = 24, 42.1%). For three participants in this group (5.3%), no subtype was specified. Five participants were treated with stimulants (Medikinet, Ritalin), 15 participants received antidepressant medication (Amitriptyline, Citalopram, Escitalopram, Elontril, Moclobemide, Venlafaxine, Vortioxetine), and three participants (5.3%) reported taking antipsychotic medication (Quetiapine) or an anxiolytic (Diazepam). Comorbidities were found in our sample of adults with ADHD. Twenty-six participants (45.6%) presented with at least one psychiatric or neurological comorbidity. Affective disorders were reported most frequently (n = 19), followed by anxiety (n = 5) and personality disorders (n = 5). Three participants in this group experienced a comorbid neurological disorder and two had a history of substance abuse.

#### 2.1.2. Control Group

Seventy-three participants randomly drawn from the student sample were allocated to the Control Group and completed all tests to the best of their ability. Twelve participants were excluded due to neurological or psychiatric comorbidities, and one participant had to be excluded due to a possible but uncertain diagnosis of ADHD. As such, the Control Group included 60 participants whose demographic data are summarized in Table 1. As expected, the Control Group reported low levels of ADHD symptoms currently and retrospectively, which differed significantly from the ADHD group.

#### 2.1.3. Simulation Group

Also drawn from the student sample, 168 participants were randomly assigned to the Simulation Group and received one of three sets of instructions: naive simulators received general instructions to feign ADHD and no additional information (n = 57), symptom-coached simulators were given the DSM diagnostic criteria of ADHD (n = 53), and fully coached simulators (n = 58) received information on both the neuropsychological assessment of ADHD and its diagnostic criteria. These three coaching conditions were summarized into one Simulation Group, as examinees intending to feign ADHD are likely to have varying levels of knowledge about ADHD and its assessment in real-life settings. Seventeen participants were excluded from this group due to failed manipulation checks. Demographic data describing the remaining 151 instructed simulators can be found in Table 1. As expected, the Simulation Group reported low levels of ADHD symptoms currently and retrospectively similar to the Control Group, which differed significantly from the ADHD group.

### 2.2. Materials

#### 2.2.1. Demographic Information

A brief demographic questionnaire was used to collect information about the participants’ demographics (e.g., age, gender, education), potential psychiatric comorbidities, medications, and ADHD diagnostic status.

#### 2.2.2. Self-Reported Symptoms of ADHD

Wender Utah Rating Scale (WURS-K). The WURS-K is the abbreviated German form of the Wender Utah Rating Scale, a self-report questionnaire used to retrospectively assess ADHD symptoms that occurred in childhood [86]. It contains 25 items, four of which are not related to ADHD and assess response tendencies. On a 5-point Likert scale ranging from 0 (= does not apply) to 4 (= strong manifestation), respondents rate how strongly various childhood behaviors applied to them. A sum score is calculated after excluding the four unrelated items, and sum scores of 30 or higher suggest clinically relevant symptoms.

ADHD Self-Report Scale (ADHS-SB). The ADHS-SB [87] is an 18-item self-report scale that examines current ADHD symptoms in adults applying DSM and ICD 10 diagnostic criteria. Participants indicate to what extent the description of symptoms applies to them on a 4-point Likert scale ranging from 0 (= does not apply) to 3 (= severe manifestation). A sum score of 18 or higher indicates clinically significant symptoms of ADHD in adulthood.

#### 2.2.3. Performance Validity

As part of the diagnostic process, all adults with ADHD took the Test of Memory Malingering (TOMM) [88]. The TOMM, a visual memory recognition test that uses a forced-choice format and floor effects to detect noncredible performance, was considered suspect if participants correctly identified fewer than 45 of 50 items on Trials 1 or 2. Applying this cut-off score, Greve, Bianchini, and Doane [89] reported a sensitivity of 56% and a specificity of 93% for the TOMM.

#### 2.2.4. Neuropsychological Performance Assessment

All participants completed the Vienna Test System’s (VTS) [90] computerized neuropsychological test battery for the assessment of cognitive functions in adult ADHD (CFADHD) [91]. The test battery was created to detect cognitive deficits among adults with ADHD in clinical practice, not for research purposes specific to this study. Due to the naturalistic setting, not all cognitive functions discussed in the literature review were evaluated on the current patient samples.

The test battery is a compilation of established, valid ability tests that assess the cognitive areas in which adults with ADHD frequently exhibit impairments, including aspects of attention and executive functions. Its norm sample included individuals aged 15 years to 85 years without neurological or psychiatric illnesses who did not take any medication known to affect the central nervous system at the time of their participation in the validation study. Sample sizes differed between individual tests, ranging from 270 to 359 individuals. Genders were similarly represented, and educational attainment spanned all educational levels.

The CFADHD is used to assess the cognitive status of adults with ADHD and to support the diagnostic process. It provides valuable information about individual cognitive strengths and weaknesses, which may aid in treatment planning and the design of compensation strategies. Objectifying subjective impairments of cognition may further increase compliance and treatment adherence [33]. However, it is not suitable as the sole source of information in the diagnostic process.

Processing Speed and Cognitive Flexibility. The Langensteinbach Version of the Trail Making Test (TMT-L) [92] was used to assess processing speed and cognitive flexibility. In Part A, the sequence of numbers 1 through 25 was displayed on the computer screen at the same time and the participants had to connect the numbers in ascending order as quickly as possible. Part B consisted of 13 numbers (1 through 13) and 12 letters (A through L) and asked participants to connect the numbers and letters alternately and in ascending order as quickly as possible. The time required for part A (in seconds) was used to assess processing speed, whereas the time required for part B was used to assess cognitive flexibility. Parts A and B were found to have internal consistency values of 0.92 and 0.81, respectively.

Selective Attention. Selective attention was measured using the Perceptual and Attention Functions—selective Attention (WAFS) [93]. In this test, participants were shown 144 geometric stimuli (triangle, circle, and square) that could become darker, lighter, or remain unchanged. Participants were instructed to respond to 30 target stimuli (i.e., a circle darkens, a circle lightens, a square darkens, and a square lightens) by pressing the response button as quickly as possible while ignoring distracting stimuli. The outcome measures recorded included the logarithmic mean of reaction times (RT) in milliseconds, variability of reaction time (RTSD; logarithmic standard deviation of reaction times), and the number of omission errors (OE) as well as commission errors (CE). The internal consistency (Cronbach’s α) was reported to be 0.95.

Working Memory. Working memory was assessed using the 2-back design of Kirchner’s [94] verbal N-Back Task (NBV) [95]. In this task, participants were shown a series of 100 consonants one by one and were instructed to press the response button if the current consonant was identical to the previous but one consonant and ignore it if it was not. The number of correct answers was recorded as the outcome measure of interest with an internal consistency (Cronbach’s) of 0.85.

Figural Fluency. To assess figure fluency, the Langensteinbach Version of the 5-point test was used (5POINT-L) [96]. This test presented participants with five symmetrically arranged dots (presented in the same pattern as the number five on a dice). The examinees were instructed to connect at least two of the dots to make as many different designs as they could in two minutes. The number of distinct patterns generated was recorded as the main outcome variable. This variable’s internal consistency (Cronbach’s alpha) was reported to be 0.86. Additionally, the number of repetitions was registered.

Task Switching. Task switching was measured using the SWITCH [97]. In this test, a variety of bivalent stimuli were shown that could be classified according to their form (triangle/circle) and brightness (gray/black). Participants were required to respond alternately in response to either of these two dimensions (triangle/circle or gray/black). The dimensions to which participants had to respond varied after every two items. Repeated items demanded a reaction based on the same dimension as the previous item, whereas switch items required a reaction based on a new dimension. Task switching accuracy, which was the difference in the proportion of right answers for switching versus repeated tasks, was the first variable of interest. Secondly, task switching speed was gauged throughout the test.

Vigilance. The Perceptual and Attention Functions were used to measure vigilance (WAFV) [98]. It included a total of 900 squares, some of which darken occasionally. Participants had to push the response button as quickly as they could in response to 50 target stimuli (squares becoming darker) while ignoring other distracting stimuli. The number of both errors of omission and commission, as well as the logarithmic mean RT and RTSD (i.e., logarithmic standard deviation of RTs) in milliseconds were recorded. The primary variables’ internal consistency (Cronbach’s alpha) was reported to be 0.96.

Response Inhibition. The Go/No-Go test paradigm was used to assess response inhibition (INHIB) [99]. Throughout this test, a series of triangles (202) and circles (48) were displayed on screen one after the other. When triangles were shown (Go trials, 80.8% of all trials), participants had to hit the response button. However, no response was necessary when circles were shown (No-Go trials, 19.2% of all trials). The occurrence of omission and commission errors was recorded, as were the RTs and the RTSD. The internal consistency (Cronbach’s alpha) was 0.83.

Interference Control. The Stroop Interference Test, first introduced in 1935, was used to evaluate interference control [100]. The form used in the present study included two baseline conditions and two interference conditions. The first baseline condition was the reading baseline condition, in which participants were shown color words (RED, GREEN, YELLOW, and BLUE) printed in gray and instructed to hit the button corresponding to the color of the word’s meaning. The second baseline condition was the naming baseline condition, in which participants were shown banners printed in four different colors (red, green, yellow, and blue) and had to click the button that matched the color of the banners. The first interference condition was the reading interference condition, in which participants were shown color words printed in mismatching ink (e.g., RED printed with green ink) and asked to press the button with the same color as the meaning of the color word while ignoring the ink. The second interference condition was the naming interference condition, which differed from the reading interference condition in that participants were instructed to press the button with the same color as the ink of the color word while ignoring its meaning. Throughout the test, participants were instructed to react as quickly as possible. Reading interference and naming interference were the variables of interest. The former was calculated by subtracting the time required for the baseline reading condition from the time required for the interference reading condition. The time required for the naming baseline condition was subtracted from the time required for the naming interference condition to calculate naming interference. The main variables’ internal consistency (Cronbach’s) was reported to be 0.97.

### 2.3. Procedure

The following assessment procedures were approved by the University of Heidelberg and the Ethical Committee Psychology (ECP) at the University of Groningen (approval number 15019-NE).

Each participant provided written informed consent and was subsequently tested individually. Adults with ADHD and neurotypical controls continued to complete all further tasks to the best of their ability. This included an extensive neuropsychological evaluation encompassing a brief history taking, self-report measures, neuropsychological performance tests, and a validity test.

Participants randomly allocated to the Simulation Group, on the other hand, received the instruction to complete the same assessment protocol as though they had ADHD. As people who aim to feign ADHD in real life likely differ in their knowledge of the disorder and the evaluation of symptom and performance validity, participants in this group were further divided into subgroups that received different levels of coaching (see Supplementary Materials Table S1). Naive simulators received a vignette that emphasized the benefits of simulating ADHD and instructions for simulating ADHD realistically but provided no information about the disorder itself. After reading the information, participants were instructed to begin simulating ADHD. In addition to the general vignette provided to naive simulators, symptom-coached simulators were given information on the DSM diagnostic criteria for ADHD. Diagnostic criteria define ADHD symptomatology and the number of symptoms required to be diagnosed with one of the ADHD subtypes. To determine whether the participant retained the information, the symptom-coached simulators had to correctly answer the first set of manipulation check questions. Only then were they allowed to begin the simulation. Lastly, the general simulation instructions, the DSM diagnostic criteria, and information on the neuropsychological assessment of ADHD were given to the fully coached simulators. Additional information included the typical procedure of a neuropsychological assessment and the commonly used assessment tools, as well as a detailed description of validity tests and the various rationales on which they are based. As a manipulation check, neither group was allowed to begin the simulation unless they correctly answered both sets of questions from the instruction check.

Completion took approximately 2 ½ h. Following the completion of the last test, all instructed simulators were asked to stop simulating ADHD and answered three questions regarding self-reported effort, subjective success in simulating the disorder, and strategies used to feign it.

### 2.4. Data Analysis

To provide context for all further analyses, we first computed summary statistics on the neuropsychological test performance and gauged the base rate of impairments across various domains. By determining which values ensured at least 90% specificity among adults with ADHD, we further derived new cut-off scores for 21 test parameters gathered by the CFADHD. Subsequently, we calculated the percentages of instructed simulators identified by these cut-off scores as well as the cut-off scores’ positive (PPV) and negative predictive values (NPV).

Data analysis was conducted in SPSS [101] and R (Version 4.2.1) [102], using the R-packages *gtsummary* (Version 1.6.1) [103], *papaja* (Version 0.1.1) [104], *tidyverse* (Version 1.3.2) [105], and *yardstick* (Version 1.1.0) [106].

## 3. Results

### 3.1. Neuropsychological Test Performance

Aggregate statistics on participants’ neuropsychological test performance can be found in Table 2. As expected, the Control Group presented with overall higher performance scores than the ADHD Group. In line with the test battery’s standardization, no more than 20% of controls presented with percentile ranks below nine on any given outcome variable. Similarly, and consistent with our expectations, an elevated percentage of—but not all—adults with ADHD demonstrated impairments on multiple tests and outcome measures.

Impairments, as defined by at least one result (i.e., any one outcome variable per test) falling below the 9th percentile, were most commonly observed in the domain of selective attention (WAFS) in both adults with ADHD (50.9%) and controls (28.3%). Impairments noted on the WAFS were most often due to an unusually high dispersion of response times in either group (WAFS RTSD; ADHD Group: 29.8%, Control Group = 20.0%), followed by high numbers of omission errors among adults with ADHD (WAFS OE; 24.6%) and commission errors among controls (WAFS CE; 10.0%). In both groups, impairments of vigilance (WAFV; ADHD Group: 43.9%, Control Group: 23.3%) and inhibition (INHIB; ADHD Group: 40.4%, Control Group = 18.3%) were second and third to those seen in selective attention. Omission errors (WAFV OE; 26.3%) and commission errors (WAFV CE; 24.6%) were the most common causes of below-average WAFV results for adults with ADHD. Both types of errors, as well as the dispersion of reaction times, showed similar rates of impairments in the Control Group (10% to 13% for WAFV OE, WAFV CE, and WAFV RTSD). Irrespective of group membership, omission errors were the driving force behind impairments of response inhibition (INHIB OE; ADHD Group: 28.1%, Control Group = 15.0%). In addition to these three tests, impairments in processing speed (TMT-A; 35.1%), interference control (STROOP; 35.1%), or flexibility (TMT-B; 21.1%) were also common among adults with ADHD.

Instructed simulators showed an overall poorer neuropsychological test performance than adults with ADHD. The percentage of participants demonstrating impairments tended to be higher in the Simulation Group than the ADHD Group and Control Group. Exceptions included the TMT-B, the number of correct responses on the NBV and the 5POINT, and the accuracy and speed measured throughout the SWITCH, where more adults with ADHD than instructed simulators presented with results below the ninth percentile.

### 3.2. CFADHD-Specific Cut-Off Scores

Cut-off scores, which ensured at least 90% specificity in our ADHD Group, are shown in Table 3.

**Figure 1:**
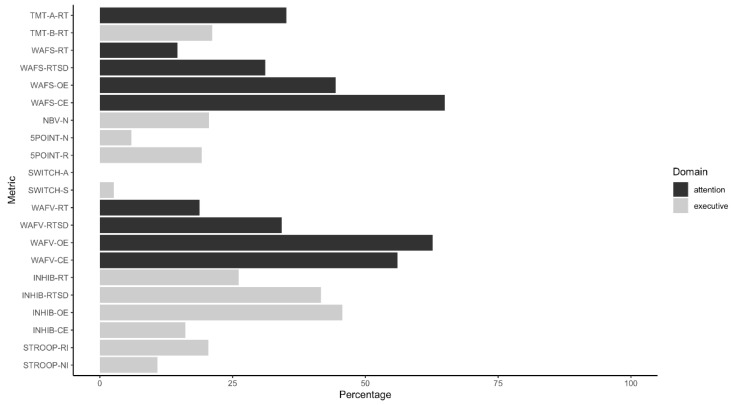
Percentage of instructed simulators detected by cut-off scores.

Figure 1 illustrates the percentage of participants in the Simulation Group who were identified by these cut-offs. Seven out of ten indicators detecting the highest percentages of the Simulation Group tapped aspects of attention (WAFS, WAFV, TMT-A); the remainder measured an executive function, namely response inhibition (INHIB). Commission errors observed on the WAFS and omission errors registered during the WAFV were most sensitive to the instructed simulation of ADHD in our sample (64.9% and 62.7% of participants detected, respectively). In both WAFS and WAFV, the number of either type of error (i.e., OE and CE) detected the highest number of instructed simulators, followed by the dispersion of response times (RTSD) and, lastly, the response times (RT) themselves. Furthermore, omission errors and the dispersion of response times registered throughout the INHIB identified 45.6% and 41.6% of instructed simulators, respectively. Response times (26.2%) and commission errors (16.1%) on the INHIB were less sensitive to feigned adult ADHD. Overall, tests tapping executive functions showed variable results, with the SWITCH detecting the smallest percentage of instructed simulators of all tests in the battery and other outcome measures (e.g., TMT-B and Reading Interference Trial of the STROOP) being mid-table. Table 4 shows the sensitivity, specificity, positive predictive values (PPV), and negative predictive values (NPV) of the cut-off scores for various base rates.

**Figure 2:**
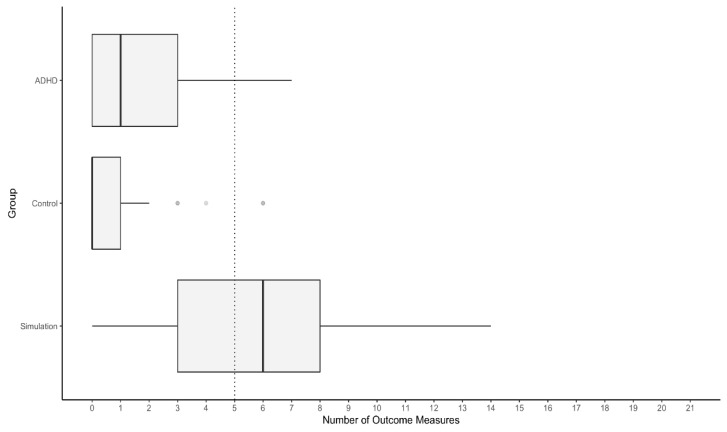
Distribution of the number of outcome measures in the suspect range. Note. The dashed line indicates the number of suspect test results that misclassify less than 10% of the ADHD Group as noncredible.

The Simulation Group showed a higher median number of test results falling into the suspect range based on the newly derived cut-off scores than the ADHD Group and Control Group. Fewer than 10% of participants with ADHD presented with five or more measured outcomes marked as suspect by the respective cut-off scores (Figure 2). In contrast, 58.9% of participants in the Simulation Group showed five or more test results in this range. Notably, data on the SWITCH and the STROOP were incomplete for four participants and two examinees missed data on the WAFV and the INHIB.

## 4. Discussion

There is broad consensus on the importance of considering symptom and performance validity when assessing adults for possible ADHD. A plethora of embedded and stand-alone, ADHD-specific, and general-purpose instruments are available to clinicians intending to follow this recommendation. Studies on these instruments have suggested an added value for a conservative approach, which requires multiple validity tests to yield suspect results for an examinee to be considered noncredible [81]. Approximately 20% of examinees have been classified as noncredible in studies following such a stringent approach, which is close to the base rate of feigned ADHD estimated by practicing clinical neuropsychologists [82]. Neuropsychological test batteries, as they are commonly used in the assessment of adult ADHD, could accommodate this approach by embedding validity indicators into their individual tests. In conducting the present study, we aimed to develop cut-off scores that could aid in the detection of non-credible performance on a neuropsychological test battery complied specifically for the assessment of ADHD. A total of eight tests tapped aspects of attention and executive functions relevant to ADHD and allowed us to provide information on the classification accuracy provided by multiple tests considered in combination.

The base rates of impairment demonstrated by neurotypical controls were in line with the tests’ standardizations. Results below the ninth percentile were more common in tests that included the highest number of outcome measures, namely those evaluating the domains of selective attention (WAFS; 28.3%), vigilance (WAFV; 23.3%), and inhibition (INHIB; 18.3%). Across all tests and assessed cognitive domains, at least 20% of adults with ADHD showed one or more results below the ninth percentile. Impairments could most commonly be observed in the domains of selective attention (WAFS; 50.9%), vigilance (WAFV; 43.9%), and inhibition (INHIB; 40.4%). Additionally, reductions in processing speed were common in our sample of adults with ADHD (TMT-A; 35.1%). The outcome variables that contributed to these impairment rates differed between tests. On the WAFS, impairments were most often due to an elevated dispersion of response times or errors of omission, whereas errors of omission and commission were most commonly below average on the WAFV.

Easier tests have been suggested to provide tendentially higher classification accuracies than their more difficult counterparts, likely as a result of the latter tapping genuine impairment e.g., [60], see [73] for a counterexample. If the base rate of impairment observed for a given test indicates its difficulty, our findings contrast with this observation: there was a strong positive association between the base rate of impairment and the percentage of instructed simulators presenting with below-average results in our sample. Put differently, tests that included at least one outcome measure with results commonly falling below the ninth percentile among adults with ADHD or their neurotypical peers, also showed the highest rates of impairment among instructed simulators. Individual outcome measures revealed more distinct profiles for each group than cognitive domains or tests. Five outcome measures demonstrated lower rates of impairment among instructed simulators than adults with ADHD (i.e., TMT-B, correct responses in a variation of the N-back task (NBV) and a computerized version of the Five-Point Test (5POINT), as well as speed and accuracy in a test of task switching (SWITCH)). The high number of omission errors on the WAFS and the WAFV, as well as frequent errors of commission on the WAFV further set the Simulation Group apart from the others. Overall, the rates of impairment in our group of instructed simulators suggested that, although the instructed simulation of adult ADHD is associated with poorer neuropsychological test performance, these effects are not uniform over the course of the assessment. This provides tentative support for the domain-specificity hypothesis and evidence against unchanging simulation efforts throughout the testing session.

The cut-off scores we derived for these 21 outcome measures painted a picture in agreement with previous studies. Although all ensured at least 90% specificity in our group of adults with ADHD, sensitivity to feigned ADHD varied significantly between the outcome variables and their respective cut-off scores. Tests measuring aspects of attention showed the highest sensitivity to the instructed simulation of ADHD, and the sensitivity of tests tapping executive functions was as broad ranging as the wide array of executive functions themselves. Tests of selective attention (WAFS) and vigilance (WAFV) detected approximately 60% of the Simulation Group, making them the most promising instruments in the present study. A variation of the Go/NoGo paradigm included in our test battery (i.e., the INHIB) correctly identified up to 45% of instructed simulators and therefore emerged as a potentially useful adjunct marker of invalid performance. As was the case for the WAFV, omission errors were most sensitive to the instructed simulation of ADHD. The comparative high sensitivity of the vigilance test included in our test battery is in line with earlier findings on the usefulness of continuous performance tests (CPTs) in the detection of invalid performance. Indeed, the sensitivity of errors of omission and commission resembled estimates reported earlier [15,43,48], whereas the sensitivity of reaction times and their variability was significantly lower than reported in studies, which identified these outcome measures as particularly promising [44,54,107]. Their ability to detect invalid performance in our sample was similar to the sensitivities reported by Ord and colleagues [51] and Scimeca and colleagues [52,108]. Notably, at the estimated base rate of noncredible performance in ADHD assessments of 20%, PPVs indicated higher classification accuracies than suggested by the sensitivity estimates. In contrast, NPVs were slightly lower than the required specificity of 90%, highlighting the importance of independent validation.

Several other tests showed lower sensitivities in the present study than previously estimated. Both versions of the TMT were less sensitive to the instructed simulation of ADHD than the detection of invalid performance described in earlier studies [43,47,61,70] and more akin to the overall lower estimates reported by Ausloos-Lozano and colleagues [69]. The sensitivity of the Stroop Tests’ Reading Interference Trial was comparable to results Shura and colleagues [47] noted for a raw-score-based cut-off value for the Word Reading Trial, yet lower than classification accuracies described elsewhere [59,60,61]. The N-back task included in the CFADHD (NBV) was less sensitive to feigned ADHD than complex memory spans to invalid performance in an assessment of adult ADHD [73]. Similarly, verbal fluency has been shown to be more sensitive to invalid performance in mixed clinical samples [61,74,75] than figural fluency in the present analogue design. A test of task switching (i.e., another executive function) which has—to the best of our knowledge—not been previously examined as a potential EVI, showed no utility in detecting the instructed simulation of ADHD.

Previous studies differed in whether raw scores, T-Scores adjusted for age, or both were considered when developing cut-off scores for EVIs. Raw scores have been reported to have favorable sensitivity, yet greater demographic diversity or formally lower educational attainment in the population under study may tip the scales in favor of T-Scores [59,61]. Indeed, raw-score derived cut-off values performed better in our sample than cut-off scores based on percentile ranks, possibly due to the restriction of standardized scores in the extreme ranges [52]. Percentiles between two and four showed marginally lower sensitivities while ensuring similarly high specificity as our raw-score-based cut-off scores. Therefore, the invalid-before-impaired paradox first posited by Erdodi and Lichtenstein [14] did not appear to apply to the cut-off scores described here, likely as a result of prioritizing high specificity in a test battery compiled specifically for ADHD assessment.

Aggregating multiple indicators, rather than interpreting individual metrices, has previously been implied as a promising approach to the evaluation of performance validity [15,16]. Although we did join multiple tests in a composite EVI (CEVI), our results suggest that the number of results in the extreme range may serve as a valuable marker of performance validity. Instructed simulators presented a higher average number of suspect results in the neuropsychological test battery than adults with ADHD and neurotypical controls. Whereas fewer than 10% of adults with ADHD showed five or more suspect results, 58% of the instructed simulators evidenced five or more outcome measures detected by the newly derived cutoff scores. As previously suggested by Erdodi and colleagues [16], applying more lenient cut-off scores to individual tests and examining the classification accuracy achieved by their combination may be worth further examination.

### Limitations

The following limitations inherent to our study may also inform future research. The limitations inherent to simulation designs, including reduced external validity, are relevant to the present study and its findings. By drawing our sample of instructed simulators from a pool of university students, we also included a population highly relevant to research on feigned ADHD. However, this recruitment procedure resulted in significant demographic differences between the groups, with the Simulation Group being significantly younger than the ADHD Group and the Control Group.

The development of embedded validity indicators based on raw scores may further produce misleading results when applied to populations that differ from the initial study sample. Indicators which were, for example, developed based on data collected in a sample of young adults may prove less accurate when applied to older adults. This concern may be alleviated, in part, by the age brackets and demographic backgrounds covered in our ADHD Group. Our sample of adults with ADHD was, however, unusually often diagnosed with the combined symptom presentation. The applicability of our cut-off scores and findings would therefore be reduced should different symptom presentations be associated with distinct cognitive profiles. Additionally, the inclusion of cases with invalid performance cannot be ruled out conclusively; even though experienced clinicians consulted a variety of instruments and sources throughout the diagnostic process, diagnoses were only made where there was objective evidence of impairment, and all participants in the current study passed an independent performance validity test. The rate of performance validity test failure (i.e., 5% of participants initially included in the ADHD Group and 6% of participants who ultimately received a diagnosis of ADHD) was lower than the estimated base rate of noncredible test performance in the evaluation of adult ADHD (i.e., 20%), likely in part due to the referral context. For example, adults being referred to specialized clinics for the clinical evaluation of ADHD may present with lower base rates of noncredible performance than college students. Not every sub-population of examinees may show a base rate of validity test failure equal to or above 20%.

Having derived our cut-off scores from raw scores bears the risk of overfitting. Cut-off scores for reaction time measures, for example, required three decimals to ensure adequate specificity, thereby potentially reducing their generalizability to other samples or settings. Lastly, a small percentage of participants missed data parts of the test battery, which may have influenced our analysis of the number of test failures. Taken together, these limitations underscore the importance of independent validation before our findings are applied in clinical practice. To counteract concerns about the external validity of analogue designs, such a validation study should preferably be conducted on a large clinical sample.

## 5. Conclusions

Impairments demonstrated by instructed simulators go beyond the neuropsychological test profiles of genuine patients in both magnitude and frequency: adults instructed to feign ADHD present with more extreme scores and higher impairment rates in most cognitive domains and tests. Simulation effort could thus be observed throughout the assessment, yet certain cognitive domains appeared more promising in detecting invalid performance than others. Selective attention, vigilance, and inhibition were most accurate in detecting the instructed simulation of adult ADHD in our sample, whereas figural fluency and task switching lacked sensitivity. Results falling between the second to fourth percentile were very rare among adults with ADHD in our sample but could more often be noted among instructed simulators. If these findings stand the test of independent validation, five or more outcome variables in this extreme range can help clinicians detect invalid performance in the neuropsychological assessment of ADHD.

## Figures and Tables

**Table 1:** Demographic data.

Characteristic	ADHD n = 57 ^1^	Control n = 60 ^1^	Simulation n = 151 ^1^	*p*-Value ^2^
Age (years)	32 (12)	20 (1)	19 (1)	<0.001
Gender (f/m)	19 (33%)/ 38 (67%)	43 (72%)/ 17 (28%)	120 (79%)/ 31 (21%)	<0.001
Education (years)	10 (1)	13 (1)	13 (1)	<0.001
ADHD Symptoms (childhood)	41 (18)	14 (9)	13 (9)	<0.001
ADHD Symptoms (adulthood)	34 (9)	11 (7)	10 (7)	<0.001

^1^ Median (MAD); n (%), ^2^ Kruskal–Wallis rank sum test; Pearson’s chi-squared test.

**Table 2:** Summary statistics on neuropsychological test performance.

	ADHD					Control					Simulation				
	Raw Score				PR < 9	Raw Score				PR < 9	Raw Score				PR < 9
Test	Median	MAD	Min	Max	%	Median	MAD	Min	Max	%	Median	MAD	Min	Max	%
TMT-A RT	20.10	3.30	12.50	40.60	35.09	14.90	1.30	10.60	24.00	0.00	20.80	6.20	11.60	151.90	35.10
TMT-B RT	32.80	7.50	15.30	96.60	21.05	20.80	3.95	13.00	43.10	0.00	33.00	9.80	13.60	529.00	19.21
WAFS RT	368	63.00	150.00	694.00	19.30	326.50	53.00	200.00	524.00	8.33	406.00	62.00	71.00	710.00	30.46
WAFS RTSD	1.25	0.08	1.12	9.33	29.82	1.21	0.05	1.11	2.80	20.00	1.36	0.06	1.14	11.86	73.51
WAFS OE	0.00	0.00	0.00	16.00	24.56	0.00	0.00	0.00	9.00	8.33	3.00	3.00	0.00	17.00	67.55
WAFS CE	3.00	1.00	0.00	49.00	15.79	2.00	1.00	0.00	10.00	10.00	10.00	5.00	0.00	87.00	77.48
NBV N	11.00	2.00	1.00	15.00	22.81	14.00	1.00	5.00	15.00	1.67	9.00	2.00	0.00	15.00	20.53
5POINT N	23.00	5.00	11.00	45.00	21.05	31.00	7.00	12.00	50.00	0.00	26.00	8.00	4.00	48.00	4.64
5POINT R	1.00	1.00	0.00	22.00	3.51	1.00	1.00	0.00	14.00	6.67	2.00	1.00	0.00	27.00	7.28
SWITCH A	0.11	2.11	−8.00	34.00	15.79	0.02	0.02	−0.05	0.13	5.00	0.03	0.04	−0.17	10.00	11.26
SWITCH S	0.24	0.15	−0.40	1.54	10.53	0.19	0.10	−0.24	0.49	3.33	0.10	0.11	−0.45	0.72	6.62
WAFV RT	464	65.00	355	662	17.54	418	51.50	247	652	6.67	548	69.00	69	751	32.45
WAFV RTSD	1.26	0.05	1.17	1.43	12.28	1.25	0.05	1.12	3.86	11.67	1.31	0.07	0.00	18.68	35.76
WAFV OE	2.00	2.00	0.00	18.00	26.32	1.00	1.00	0.00	11.00	10.00	9.00	4.00	0.00	25.00	82.12
WAFV CE	2.00	1.00	0.00	17.00	24.56	1.00	1.00	0.00	8.00	13.33	7.00	4.00	0.00	298.00	76.82
INHIB RT	0.28	0.03	0.20	0.51	7.02	0.26	0.03	0.21	0.41	1.67	0.31	0.05	0.21	0.56	25.17
INHIB RTSD	0.10	0.03	0.05	3.25	21.05	0.08	0.02	0.04	0.33	3.33	0.15	0.05	0.04	1.83	51.66
INHIB OE	4.00	3.00	0.00	33.00	28.07	2.00	2.00	0.00	46.00	15.00	15.00	10.00	0.00	121.00	65.56
INHIB CE	12.00	4.00	1.00	35.00	19.30	12.00	3.00	1.00	32.00	8.33	19.00	5.00	2.00	43.00	43.05
STROOP RI	0.18	0.10	−0.03	0.88	31.58	0.12	0.04	0.00	0.51	8.33	0.22	0.12	−0.71	2.31	33.11
STROOP NI	0.11	0.08	−0.12	0.85	10.53	0.11	0.04	0.00	1.16	0.00	0.10	0.06	−0.29	1.00	13.25

MAD = Median Absolute Deviation, TMT = Trail Making Test, WAFS = Perceptual and Attention Functions—selective Attention, NBV = N-Back Task, 5POINT = 5-Point Test, SWITCH = test of task switching, WAFV = Perceptual and Attention Functions—vigilance, INHIB = adaptation of Go/No-Go paradigm, STROOP = adaptation of color and word interference test, RT = Response Time, RTSD = Dispersion of Response Times, OE = Omission Errors, CE = Commission Errors, N = Number of correct responses, R = number of Repetitions, A = Accuracy, S = Speed, RI = Reading Interference, NI = Naming Interference.

**Table 3:** Cut-off scores.

Test	Indicator	Cut-off Score
TMT-A	RT	≥27.1
TMT-B	RT	≥54.6
WAFS	RT	≥532
WAFS	RTSD	≥1.43
WAFS	OE	≥4
WAFS	CE	≥8
NBV	N	≤6
5POINT	N	≤12
5POINT	R	≥4
SWITCH	A	≥11
SWITCH	S	≥0.54
WAFV	RT	≥636
WAFV	RTSD	≥1.35
WAFV	OE	≥7
WAFV	CE	≥7
INHIB	RT	≥0.364
INHIB	RTSD	≥0.165
INHIB	OE	≥18
INHIB	CE	≥27
STROOP	RI	≥0.448
STROOP	NI	≥0.323

TMT = Trail Making Test, WAFS = Perceptual and Attention Functions—selective Attention, NBV = N-Back Task, 5POINT = 5-Point Test, SWITCH = test of task switching, WAFV = Perceptual and Attention Functions—vigilance, INHIB = adaptation of Go/No-Go paradigm, STROOP = adaptation of color and word interference test, RT = Response Time, RTSD = Dispersion of Response Times, OE = Omission Errors, CE = Commission Errors, N = Number of correct responses, R = number of Repetitions, A = Accuracy, S = Speed, RI = Reading Interference, NI = Naming Interference.

**Table 4:** Sensitivity, Specificity, and Positive (PPV) and Negative Predictive Values (NPV).

			Base Rate									
			10%		20%		30%		40%		50%	
Metric	Sensitivity	Specificity	PPV	NPV	PPV	NPV	PPV	NPV	PPV	NPV	PPV	NPV
TMT-A RT	35.10	91.23	30.78	92.67	50.01	84.90	63.17	76.63	72.73	67.83	80.01	58.43
TMT-B RT	21.19	91.23	21.16	91.24	37.65	82.24	50.87	72.98	61.69	63.46	70.73	53.65
WAFS RT	14.57	91.23	15.58	90.58	29.34	81.03	41.58	71.36	52.55	61.56	62.42	51.64
WAFS RTSD	31.13	91.23	28.28	92.26	47.01	84.12	60.33	75.55	70.29	66.52	78.01	56.98
WAFS OE	44.37	92.98	41.26	93.77	61.25	86.99	73.04	79.59	80.83	71.49	86.34	62.57
WAFS CE	64.90	91.23	45.12	95.90	64.91	91.23	76.02	85.85	83.14	79.59	88.09	72.22
NBV N	20.53	91.23	20.64	91.18	36.91	82.12	50.08	72.82	60.94	63.26	70.06	53.44
5POINT N	5.96	96.49	15.88	90.23	29.81	80.41	42.13	70.54	53.11	60.62	62.94	50.64
5POINT R	19.21	91.23	19.57	91.04	35.37	81.87	48.41	72.49	59.34	62.88	68.65	53.03
SWITCH A	0.00	90.57	0.00	89.07	0.00	78.37	0.00	67.88	0.00	57.60	0.00	47.52
SWITCH S	2.65	90.57	3.03	89.33	6.56	78.82	10.74	68.46	15.77	58.25	21.92	48.19
WAFV RT	18.79	91.23	19.23	91.00	34.88	81.80	47.87	72.39	58.82	62.76	68.18	52.91
WAFV RTSD	34.23	91.23	30.24	92.58	49.38	84.73	62.58	76.40	72.23	67.54	79.60	58.11
WAFV OE	62.67	91.23	44.25	95.65	64.11	90.72	75.38	85.08	82.65	78.57	87.72	70.96
WAFV CE	56.00	91.23	41.50	94.91	61.48	89.24	73.23	82.87	80.97	75.67	86.46	67.46
INHIB RT	26.17	91.23	24.90	91.75	42.73	83.17	56.12	74.25	66.55	64.96	74.90	55.27
INHIB RTSD	41.61	91.23	34.52	93.36	54.25	86.21	67.03	78.47	75.98	70.09	82.59	60.97
INHIB OE	45.64	91.23	36.63	93.79	56.53	87.03	69.04	79.66	77.62	71.57	83.88	62.66
INHIB CE	16.11	94.74	25.38	91.04	43.35	81.87	56.74	72.49	67.11	62.88	75.37	53.04
STROOP RI	20.41	91.23	20.54	91.16	36.77	82.09	49.93	72.79	60.80	63.23	69.94	53.41
STROOP NI	10.88	91.23	12.12	90.21	23.68	80.37	34.72	70.49	45.27	60.56	55.37	50.59

TMT = Trail Making Test, WAFS = Perceptual and Attention Functions—selective Attention, NBV = N-Back Task, 5POINT = 5-Point Test, SWITCH = test of task switching, WAFV = Perceptual and Attention Functions—vigilance, INHIB = adaptation of Go/No-Go paradigm, STROOP = adaptation of color and word interference test, RT = Response Time, RTSD = Dispersion of Response Times, OE = Omission Errors, CE = Commission Errors, N = Number of correct responses, R = number of Repetitions, A = Accuracy, S = Speed, RI = Reading Interference, NI = Naming Interference.

## Data Availability

The data that support the findings of this study are available from the corresponding author, A.B.M.F., upon reasonable request.

## Supplementary Materials

The following supporting information can be downloaded at: https://www.mdpi.com/article/10.3390/ijerph20054070/s1, Table S1: Information provided to instructed simulators.
