# Supplementary material for: Feigning Adult ADHD on a Comprehensive Neuropsychological Test Battery: An Analogue Study
Source: Int J Environ Res Public Health. 2023 Feb 24;20(5):4070. doi: 10.3390/ijerph20054070 (PMC10001580; doi:10.3390/ijerph20054070)
Supplement: Supplementary file 1 [file ijerph-20-04070-s001.zip › ijerph-2181991-supplementary.pdf]

International Journal of
Environmental Research and Public Health

Supplementary Material

Table S1. Instructions and information provided to instructed simulators.

**Instruction: Simulation of Attention Deficit Hyperactivity Disorder in adults**
Imagine you read about a condition called ADHD in adults. Your interest is sparked and you start researching the literature and the Internet about this condition.<br>You find out that a diagnosis of ADHD could benefit you in many ways. For example some employees and universities provide special equipment for adults with ADHD, such as computers and electronic personal organizers. People with ADHD are further entitled to flexible working hours and to less strict deadlines.<br>You also read that certain medicines, so-called stimulants, which are usually prescribed to ADHD patients, have a strong positive effect. These medicines can be taken occasionally and result in a significant rise in the ability to concentrate, resulting in increased efficiency, without strong side effects. You find out that it helps to be more organized and to come to grip with things. This would not only help you to do your work better but would also give you more energy after work for leisure activities. Some of the articles you read even claim that patients with ADHD occasionally receive milder legal punishments for their actions.<br>You are convinced that a diagnosis of ADHD would benefit you a lot, and you decide to simulate adult ADHD.<br>You go to see a doctor or psychologist. You report your alleged symptoms and you are trying to convince your doctor that you are suffering from ADHD. You want to come across as believably affected by the disorder in order to gain all the benefits, therefore you have to do all you can to act exactly like a patient with ADHD.<br><br>Please act precisely as if you had ADHD! Try to be as convincing as possible! You are trying to convince me that you have ADHD, please also be careful not to overdo it!<br><br>Your test results will be analyzed after the study and compared to data of genuine patients with ADHD.

Instruction: Simulation of Attention Deficit Hyperactivity Disorder in adults

The attention- deficit hyperactivity disorder is a developmental disorder with childhood onset. 3% to 5% of children are affected worldwide. 3 different core symptoms are distinguished in ADHD:

1) Inattention
2) Hyperactivity
3) Impulsivity

Recent studies show that adults can be affected by the disorder as well. It is estimated that 40 % to 60 % of children with ADHD still suffer from the disease later on in adulthood.

However the symptoms change slightly. Adults with ADHD show mainly symptoms of **inattention** and **impulsivity**. Symptoms of hyperactivity are less pronounced and shift towards an "**inner unrest**".

Several criteria have to be fulfilled in order to get diagnosed with ADHD. The criteria are tested behaviorally and are judged based on the self- evaluation of the patient and the assessment of the specialist.

There are 9 criteria of inattention of which at least 5 have to be fulfilled:

Symptoms of Inattention

The patient

1) Often does not give close attention to details or makes careless mistakes in work, or other activities.
2) Often has trouble keeping attention on tasks at work or other activities (eg. reading, watching TV)
3) Often does not seem to listen when spoken to directly.
4) Often does not follow instructions and fails to finish duties in the workplace.
5) Often has trouble organizing activities (e.g. planning a holiday).
6) Often avoids, dislikes, or doesn't want to do things that take a lot of mental effort for a long period of time.
7) Often displaces important objects (e.g. keys, wallet, tools, glasses).
8) Is often easily distracted.
9) Is often forgetful in daily activities (e.g. meetings, telephone calls, appointments).

There are 9 criteria of hyperactivity/ impulsivity of which at least 5 have to be fulfilled:

Symptoms of Hyperactivity

The patient

1) Often fidgets with hands or feet or squirms in seat.
2) Has difficulties sitting still (e.g. in the cinema or theater).
3) Often has feelings of inner restlessness.
4) Often has difficulty engaging in (leisure) activities quietly.
5) Is often "on the go" or often acts as if "driven by a motor".

Symptoms of Impulsivity

The patient

6) Often has difficulties to wait until others have finished talking.
7) Often has difficulties to wait until it is his/her turn (e.g. in the supermarket).
8) Often interrupts or intrudes on others.
9) Often talks excessively.

Depending on the range that the symptoms are corresponding to 3 subtypes of ADHD can be distinguished:

1) Primarily Inattentive Subtype: The individual fulfills criteria of inattention but not of hyperactivity or impulsivity.
2) Primarily Hyperactive/Impulsive: The individual fulfills criteria of hyperactivity/ impulsivity but not of inattention.
3) Combined Subtype: The individual has symptoms of inattention as well as impulsivity and hyperactivity.

The neuropsychological assessment of patients with attention deficit hyperactivity disorder

The neuropsychological assessment

The neuropsychological assessment takes about one to three hours, depending on its purpose and the assessment tools applied. The clinician, usually a psychologist, invites the person to a face-to-face appointment. Group-sessions are relatively rare.

The patient is first asked to describe experienced problems and behavior (subjective assessment). This is done by either asking open questions, using interviews (set of structured questions), or by means of questionnaires and rating scales.

Subsequently, and objective assessment of cognitive functions is often performed (neuropsychological tests). Testing procedures include partially computerized tasks to assess abilities of attention, concentration, distractibility, impulsivity, memory and problem solving ability (such as planning and thinking etc.). A single test usually takes 3 to 10 minutes, with a few exceptions taking up to 25 minutes (e.g. sustained attention tests).
Most tests of attention are designed similarly. Stimuli are displayed consecutively on a screen. The participant is asked to focus on a predefined set of stimuli and to react to the presentation of these target stimuli as quickly as possible via a button press. Other, distracting stimuli have to be ignored. Several variables are usually measured such as reaction times, variability of reaction times and the number of errors.
In tests for memory, a number of verbal or figural stimuli are presented in several learning trials. The participant is required to memorize as many stimuli as possible. The memory performance is measured by recall or recognition tests which are performed after a certain retention interval at a later time of the assessment.

The clinician makes a concluding clinical evaluation by integrating all the acquired information: the behavioral observation, the information from the clinical interview, the information of the questionnaires as well as the information from the neuropsychological tests. It is the task of the clinician to put all the acquired information into a context and to come to a final clinical evaluation of the presence of ADHD.

Typical behavior of adults with ADHD

Many patients with ADHD have problems in domains of attention and concentration. Patients with ADHD are usually distractible, that means they ask questions repeatedly during a conversation, often have problems understanding instructions in the first place and often miss details in conversations or when reading. Memory problems, such as forgetfulness or inability to store information in memory, are also often observed in patients with ADHD.
It can be seen that patients with ADHD need more time to complete tasks than people without ADHD. This can be related to problems in attention, or to problems of structuring and planning things. It can also be seen that patients with ADHD lose their interest in things rather quickly.
Impulsive behavior of patients with ADHD frequently leads to thoughtless, premature responses. Patients with ADHD often regret these behaviors soon after. Some adults with ADHD also show the typical behavior of children with ADHD such as motor restlessness. Even though this is relatively rare in adult patients as compared to children, some patients exhibit motoric unrest and fidgeting when sitting on a chair.

**The neuropsychological assessment of patients with attention deficit hyperactivity disorder**
**Assessment of noncredible performance (detection of simulated ADHD)**<br><br>A considerable number of individuals feign symptoms to receive a diagnosis of ADHD. These people are motivated by incentives such as receiving stimulant drugs, financial compensation, or academic accommodations at educational institutes (e.g. universities).<br>There are a wide range of tests that were specifically designed for the detection of feigned cognitive dysfunctions. Some of these tests, for example, appear to be difficult, but are in fact easy to accomplish for most individuals, including individuals with severe impairments. Other tests compare the individual performance with the performance level that is expected if answers are given randomly (chance level). If the person's performance is below chance level, then noncredible symptom report is very likely. Furthermore, some tests also consider the consistency of the effort and performance over time. Inconsistent performance is also indicative for noncredible performance.<br>In order to avoid the detection of feigning, you have to watch out for tests measuring your credibility! Do not fake symptoms on easy tests which could be successfully accomplished by healthy individuals and patients. Take also care not to perform below chance level on any of the tests applied. Furthermore, try to be consistent in your strategies and effort to feign aADHD. Try to be convincing in believably feigning aADHD!
